# Distribution of Hydrothermal Alvinocaridid Shrimps: Effect of Geomorphology and Specialization to Extreme Biotopes

**DOI:** 10.1371/journal.pone.0092802

**Published:** 2014-03-27

**Authors:** Anastasia A. Lunina, Alexandr L. Vereshchaka

**Affiliations:** Laboratory of structure and dynamics of plankton communities, P.P. Shirshov Institute of Oceanology of Russian Academy of Sciences, Moscow, Russia; Fenton, University of Western Ontario, Canada

## Abstract

The aim of this study is to review of our knowledge about distribution of recently known species of vent shrimps and to analyze factors influencing distribution patterns. Analyses are based upon (1) original material taken during eight cruises in the Atlantic Ocean (a total of 5861 individuals) and (2) available literature data from the Atlantic, Pacific, and Indian Oceans. Vent shrimps have two patterns of the species ranges: local (single vent site) and regional (three - six vent sites). Pacific species ranges are mainly of the local type and the Atlantic species ranges are of the regional type. The regional type of species ranges may be associated with channels providing easy larval dispersal (rift valleys, trenches), while the local type is characteristic for other areas. Specialization of a shrimp genus to extreme vent habitats leads to two effects: (1) an increase in the number of vent fields inhabited by the genus and (2) a decrease of species number within the genus.

## Introduction

The discovery of hydrothermal vents along the Galápagos Ridge in 1977 [1] has stimulated an increasing research effort examining the diversity, ecology, physiology, and biogeography of vent organisms, as well as new avenues of research into the origins of life on Earth [2]. Unusual characteristics of deep-sea vents compared with other deep-sea habitats, coupled with the ephemeral nature of hydrothermal circulation, may have important implications for deep-sea biology [3]. Decades of exploration have revealed numerous vent sites and faunal assemblages at many mid-ocean ridges and back-arc basins. As the global biogeography of vent organisms has been elucidated, separate biogeographic provinces have been erected for the shallow and deep Atlantic, the East Pacific, the North East Pacific, West Pacific back-arc basins, and the Indian Ocean [4]. Recent studies have modified this general scheme, proving existence of a single province for the Atlantic, a single province for the North West Pacific, a single province for the South West Pacific and Indian Ocean, and a separation of the North East Pacific, North East Pacific Rise, and South East Pacific Rise [5]. However, there are some shortcomings in the methodology of Bachraty et al. [5], some of which have been addressed by Rogers et al. [3].

At the same time, several attempts have been made to understand which factors may affect the observed distributional patterns of vent biota. The effects of spreading rate and geomorphology of the mid-ocean ridge axis have been proposed to be among these factors [6]. We assume that some of patterns will become clearer and much more visible if we revise and analyze the global distribution of a selected taxonomic group, including species with similar morphological, physiological, biochemical, and reproductive features. This group should be widely distributed and have numerous species to provide statistically significant conclusions. One potential group for such study is the shrimp family Alvinocarididae.

Alvinocaridid shrimps (Caridea: Alvinocarididae) represent the key elements of hydrothermal communities of several vent fields on the Mid-Atlantic Ridge (MAR), and they are members of the hydrothermal communities in other areas of the oceans [7]. We know that numerous species occur in the Atlantic, Pacific, and Indian Oceans, with new species described during the last 10 years [8]–[18]. Despite the importance and visibility of the group, no recent review of its composition, distribution, and spatial/ecological biogeography is available.

Even among the severe conditions of the deep sea (elevated pressure, complete absence of light), the environments of hydrothermal vents may be considered extreme, with unique physical and chemical properties such as high and rapidly changing temperature (from 2–4 °C to 400 °C), acidic pH, toxic heavy metals, an hydrogen sulfide [19]–[20]. Vent shrimp genera show numerous adaptations to vent habitats. These adaptations are fewer and less conspicuous in the genus *Alvinocaris*, which is similar to usual deep-sea shrimps, and numerous and prominent in the genus *Rimicaris* [21]–[23]. Specialization to vent habitats implies morphological adaptations (highly specialized mouthparts covered with soft setae, enlarged branchial chambers formed by the carapace, appearance of dorsal organ – [21]–[23] and adaptations reflected in life cycles [24]. Specialization to hydrothermal conditions increases in the string *Alvinocaris* - *Opaepele* - *Chorocaris* - *Mirocaris - Rimicaris* [23]–[24]. Recently the family is ripe for review of its internal phylogeny.

In this paper we summarize original data about composition and distribution of Alvinocaridid vent shrimps and put them in the context of relevant general literature information. Further we try to (1) reveal general patterns of vent shrimp distribution, (2) understand factors determining the species distribution and (3) estimate role of specialization to extreme biotopes in the composition and distribution of vent shrimp genera.

## Material and Methods

**Figure 1:**
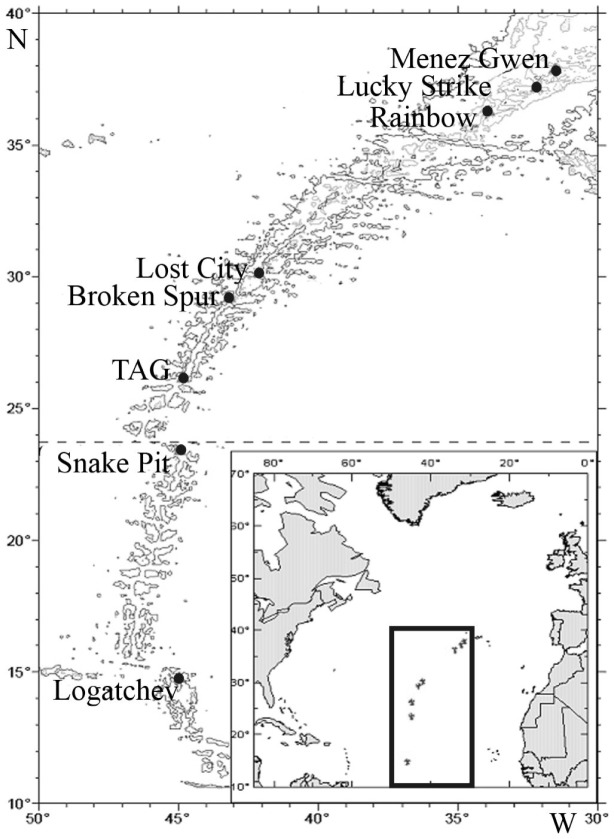
Map of the Atlantic hydrothermal vent fields visited during collection of the original material. Isobaths 500 m, 1000 m, 2000 m, and 3000 m are shown.

Original material was taken along the Mid-Atlantic Ridge during eight cruises of R/V “Akademik Mstislav Keldysh” with use of two deep-sea manned submersibles "Mir–1" and "Mir–2" (Fig. 1, Table 1). Seven vent fields were investigated during 1994-2005, including Menez Gwen (37.8417 N, 31.525 W), Lucky Strike (37.2933 N, 32.2733 W), Rainbow (36.23 N, 33.902 W), Broken Spur (29.17 N, 43.1717 W), TAG (26.1367 N, 44.8267 W), Snake Pit (23.3683 N, 44.95 W) and Logatchev (14.752 N, 44.9785 W). No specific permissions were required for field studies for all locations. The field studies did not involve endangered or protected species.

**Table 1:** Vent shrimps, original collections during 34–41 Cruises of R/V “Akademik Mstislav Keldysh”, submersibles “Mir”, Atlantic Ocean.

Hydrothermal field	*Rimicaris exoculata*		*Alvinocaris markensis*	
Hydrothermal field	Date	No of individuals	Date	No of individuals
Menez Gwen		-	01.03.1997	1
Lucky Strike		-	11.06.2002	4
Rainbow	25.10.1998	138	25.10.1998	21
	17.07.2002	40	18.07.2002	12
	17.07.2002	27	03.09.2005	110
	18.07.2002	47	-	-
	19.07.2002	61	-	-
	02.09.2005	79	-	-
	03.09.2005	68	-	-
Broken Spur	03.09.1996	75	6–8.09.1996	4
	04.09.1996	527	01.06.2002	8
	08.09.1996	49	25.08.2005	98
	08.09.1996	299	-	-
	01.07.2002	330	-	-
	01.07.2002	35	-	-
	29.09–01.10.1994	13	-	-
TAG	24.09.1994	330	26–27.06.2002	1
	24.09.1994	199	-	-
	25.06.2002	401	-	-
	26.06.2002	45	-	-
	27.06.2002	474	-	-
	17.09–22.09.1994	21	-	-
Snake Pit	20.06.2002	500	22.06.2002	22.06.2002
	20.06.2002	190	12.08.2003	12.08.2003
	21.06.02	100	-	-
	22.06.2002	82	-	-
	12.08.2003	147	-	-
	12.08.2003	211	-	-
	12.08.2003	232	-	-
	13.08.2003	65	-	-
Logachev	18.11.1998	461	01.02.1995	1
Logachev	-	-	27.07.1998	8

Faunal composition, micro-scale distribution, behavior and population structure of shrimps in various vent microbiotopes were thoroughly investigated. Shrimps were collected using baited traps and submersible suction samplers. Immediately after retrieval all specimens were sorted, measured, and fixed in 80% alcohol. Measurements follow established methods for shrimp morphological description [25]. Type material is deposited in the Zoological Museum, Moscow, and the Oxford University Museum of Natural History (OUMNH). A total of 5861 individuals of vent shrimps were analyzed.

Analysis of shrimp distribution was made with use of original and all available literature data (see references in Table 2) including original descriptions. Detailed description of material and discussion of the species status may be found in [17], [26]–[27].

**Table 2:** Recently known hydrothermal vent and seep shrimp species.

Species	Region	Habitat	Coordinates	Depth	Authors
*Alvinocaridinides formosa*	**Taiwan**	Gueishandao, Yilan County	24°51.231′N, 121°59.204′E	252–275 m	[16]
*Alvinocaris alexander*	**New Zealand, Kermadec Ridge**	Rumble V Seamount	36°08.27–35 S 178°11.74 E;	730–415 m	[14]
		Brothers Caldera	34°52.89 S 179°03.76 E	1346–1196 m	
*Alvinocaris brevitelsonis*	**Okinawa Trough**	Minami-Ensei Knoll	28°23.35'N, 127°38.38'E	705 m	[50]
*Alvinocaris chelis*	**Taiwan**	Gueishandao, Yilan County	24°49–51N 121°59–122°0′E	300–252 m	[16]
*Alvinocaris dissimilis*	**Okinawa Trough**	Minami-Ensei Knoll	28°23.35'N, 127°38.38'E	705 m	[10]
*Alvinocaris komaii*	**South-West Pacific: Lau**	Kilo Moana	20°9′S, 176°12′E	2620 m	[15]
		Tow Cam	20°19′S, 176°8′E	2700 m	
		ABE	20°45′S, 176°11′E	2145 m	
*Alvinocaris longirostris*	**Sagami Bay**	Off Hatsuchima site (cold seep)	35°00′ N; 139°14′E	∼1100 m	[28]–[29]
	**Okinawa Trough**	Iheya Ridge	27°32.70′N, 126°58.20′E	1360 m	[51]
		Hatoma Knoll	24°51′N; 123°50.4′E	∼1950 m	[52]
		Miname-Ensei Knoll	28°23.4′ N; 127°38.4′E	∼700 m	
	**New Zealand, Kermadec Ridge**	Brothers Caldera	34° 51-53′S 179° 3-4′E	1850–1196 m	[9]
*Alvinocaris lusca*	**Galapagos Rift:**	Rose Garden area	00°48.15′N, 86°13.29′W	2450 m	[53]
	**East Pacific Ridge**	9°N site,	09°50.3′N, 104°17.4′W	2520 m	[54]–[55]
*Alvinocaris markensis*	**Mid-Atlantic Ridge**	Lucky Strike	37° 17.598'N 32° 16.398'W	1600–1740 m	[56]
		Rainbow	36° 13.800'N33° 54.120'W	2270–2320 m	
		Broken Spur	29° 10.200'N 43° 10.302'W	3100 m	[57]
		TAG	26° 8.202'N 44° 49.602'W	3436–3670 m	[21]
		Snake Pit	23° 22.098'N 44° 57.000'W	3450–3500 m	[55]
		Logatchev	14° 45.120'N 44° 58.710'W	2925–3050 m	[54]
*Alvinocaris niwa*	**New Zealand: Kermadec Ridge**	Brothers Caldera	34°52.89–52.87′S 179°3.76–3.21′E;	1346–1196 m	[9]
		Rumble V Seamount	36°8.63–8.57′S, 178°11.77–11.50′E;	877–655 m	
*Alvinocaris williamsi*	**Mid-Atlantic Ridge**	Menez Gwen	37° 50.502'N 31° 31.500'W	840–865 m	[58]
*Chorocaris chacei*	**Mid-Atlantic Ridge**	Moytirra	45° 28.998'N 27° 51.000'W	3000 m	[59]
		Lucky Strike	37° 17.598'N 32° 16.398'W	1600–1740 m	[60]
		Broken Spur	29° 10.200'N 43° 10.302'W	3100 m	
		TAG	26° 8.202'N 44° 49.602'W	3436–3670 m	
		Snake Pit	23° 22.098'N 44° 57.000'W	3450–3500 m	
		Logatchev	14° 45.120'N 44° 58.710'W	2925–3050 m	
		Ashadze	12° 58.398'N 44° 51.798' W	4080 m	[61]
*Chorocaris paulexa*	**East Pacific Rise**	17 37'S, EPR, Homer Vent Site	17°37.220′S, 113°15.123′W	2596 m	[11]
		Rapa Nui vent field, Brandon vents	21°33.7′S 114°17.9′W	3640 m	
*Chorocaris vandoverae*	**Western Pacific Ocean,Mariana Back-Arc Spreading Center**	Alice Springs vent field;	18°12.599′N, 144°42.431′E;	3660 m	[55]–[56], [62]
*Nautilocaris saintlaurentae*	**North Fiji Basin**	White Lady site	16°59.50′S, 173°55.47′E,	2000 m	[63]
	**Lau Basin**	Vaï-Lili site	22°13′S, 176°38′E	1750 m	
*Opaepele loihi*	**Pacific Ocean, Hawaii**	Loihi Seamount;	18°55′N, 155°16′W	980 m	[64]
	**Mariana Arc**	NW Rota-1 Volcano	14°36.0′N, 144°46.5′E	530 m	[65]
	**Philippine Sea Plate**	Nikko Seamount	23° 4.856′ N 142° 19.512′ E	456 m	[66]
*Opaepele susannae*	**South Mid-Atlantic Ridge**	Semenov	13° 30.822'N 44° 57.780'W	2440 m	[67]
		Mephisto	04°47.834S, 12°22.593W	3045 m	
		Turtle Pits	04°48.57 S, 12°22.41 W	2998 m	[13]
		Sisters Peak	04°48.188S, 12°22.301W	2986 m	
		Lilliput	9° 33.000' S 13° 10.800'W	1500 m	
*Opaepele vavilovi*	**Mid-Atlantic Ridge**	Broken Spur	29.1700 N 43.1717 E	3100 m	[17]
*Rimicaris exoculata*	**Mid-Atlantic Ridge**	Moytirra	45° 28.998'N 27° 51.000'W	3000 m	[59]
		Lucky Strike (very low abundance)	37° 17.598'N32° 16.398'W	1600–1740 m	[10]
		Rainbow	36° 13.800'N 33° 54.120'W	2270–2320 m	[60]
		Broken Spur	29° 10.200'N 43° 10.302'W	3100 m	
		TAG	26° 8.202'N 44° 49.602'W	3436–3670 m	
		Snake Pit	23° 22.098'N 44° 57.000'W	3450–3500 m	
		Logatchev	14° 45.120'N 44° 58.710'W	2925–3050 m	
		Ashadze	12° 58.398'N 44° 51.798' W	4080 m	[61]
	**South MAR**	Mephisto	04°47.834S, 12°22.593W	3045 m	
*Rimicaris kairei*	**Central Indian Ridge, Rodriguez Triple Junction**	Kairei Field;	25°19.16′S, 70°02.40′E;	2454 m	[8]
		Edmond vent field	23°52.68′S, 69°35.80′E	3290–3320 m	[68]
		Dodo hydrothermal field	18°20.19S, 65°17.99E;	2745 m	[69]
		Solitaire hydrothermal field	19°33.413S, 65°50.888E	2606 m	
	**SW Indian Ridge**	Dragon	37° 46.998'S 49° 39.000'W	2785 m	Copley J., pers. comm.
*Rimicaris hybisae*	**Mid-Cayman Spreading Centre**	Beebe	18° 32.688'N 81° 43.170'W	4960 m	[18]
		Von Damm	18° 22.596'N 81° 47.832'W	2300 m	
*Shinkaicaris leurokolos*	**Okinawa Trough**	Minami-Ensei Knoll	28°23.35'N, 127°38.38'E	705 m	[50]
*Mirocaris fortunata*	**Mid-Atlantic Ridge**	Moytirra	45° 28.998'N 27° 51.000'W	3000 m	[59]
		Menez Gwen	37° 50.502'N 31° 31.500'W	840–865 m	[70]
		Lucky Strike	37° 17.598'N32° 16.398'W	1600–1740 m	
		Rainbow	36° 13.800'N 33° 54.120'W	2270–2320 m	
		Broken Spur	29° 10.200'N 43° 10.302'W	3100 m	
		TAG	26° 8.202'N 44° 49.602'W	3436–3670 m	
		Snake Pit	23° 22.098'N 44° 57.000'W	3450–3500 m	
		Logatchev	14° 45.120'N 44° 58.710'W	2925–3050 m	
		Ashadze	12° 58.398'N 44° 51.798' W	4080 m	[61]
	**South MAR**	Turtle Pits	04°48.57 S, 12°22.41 W	2998 m	
*Mirocaris indica*	**Central Indian Ridge, Rodriguez Triple Junction:**	Kairei Field	25°19.2′S, 70°02.4′E	2422 m	[12]
		Edmond Field	23°52.7 ‘S, 69°35.8′E,	3300 m	
		Solitaire hydrothermal field	19°33.413S, 65°50.888E	2606 m	[69]
	**SW Indian Ridge**	Dragon	37° 46.998'S 49° 39.000'W	2785 m	Copley J., pers. comm.

As differences in sampling efforts may affect current records of species' distributions, we analyzed the InterRidge database including all recently recorded active vent sites (http://irvents-new3.whoi.edu). Table 3 illustrates the number of explored active vent sites within major geographic regions and the number of sites within those areas where Alvinocaridid shrimps have been recorded. To examine the possible correlation between these two numbers, we used the Spearman correlation coefficient. For a sample of size *n* and difference *d* coefficient ρ is computed from these: ρ = 1−(6Σ*d^2^/n(n^2^*−1)). All maps were created with use of the CorelDraw (styled CorelDRAW) vector graphics editor, version X6, graphics were made with use of Microsoft Excel spreadsheet application, version 14.0.

**Table 3:** 

Region	Number of sites explored	Number of sites with shrimp records	Share of active vents inhabited by shrimps	Depth range, m	Sites explored
Southern Ocean	3	0	0.00	45–270	ESR; E2, Adventure Caldera, Kemp Caldera
Northwest Atlantic	7	2	0.29	1–4960	Champagne Hot Springs, Kick'em Jenny submarine volcano, Montserrat Volcano, Beebe, Europa, Von Damm, Don Joao de Castro Bank
Mid-Atlantic Ridge	22	11	0.50	350–4200	Ashadze, Ashadze 2, Broken Spur, Bubbylon, Evan, Logatchev, Logatchev 2, Lost City, Lucky Strike, Menez Gwen, Menez Hom, Moytirra, Rainbow, Saldanha, Semyenov, Snake Pit, TAG, Steinaholl Vent Field, Baily's Beads, Lilliput, MAR; 4 48'S, Nibelungen
Indian Ocean	6	5	0.83	1600–3320	Aden, Dodo Field, Edmond Field, Kairei Field, Solitaire Field, SWIR Area A
Central Pacific	5	2	0.40	150–4800	Loihi Seamount, Bounty Seamount, Macdonald Seamount, Teahitia vents, Vailulu'u Seamount
Galapagos Rift	6	1	0.17	1640–2700	Calyfield, Galapagos Mounds, Iguanas-Pinguinos, Navidad, Precious Stone Mountain, Rose Garden
Juan de Fuca Ridge	20	0	0.00	1540–3460	Axial Volcano; ASHES, Axial Volcano; CASM, Axial Volcano; South Rift Zone, Baby Bare Seamount, Central Cleft; off-axis, East Blanco Depression, Floc, Flow, High-Rise Field, Main Endeavour Field, Middle Valley; Dead Dog Vent Field, Middle Valley; ODP Mound, Mothra Field, North Cleft; high temperature, North Cleft; low temperature, Not Dead Yet, Salty Dawg Field, Sasquatch Field, Source, South Cleft
Kermadec Arc	9	2	0.22	130–1800	Brothers volcano, Clark volcano, Giggenbach volcano, Healy volcano, Macauley Caldera, Rumble III volcano, Rumble V volcano, Vulkanolog, Wright volcanic center
Lau Basin	17	4	0.24	1200–2700	ABE, CDE, CLSC; A3, Hine Hina, Kilo Moana, Kulo Lasi, Maka, Mariner, Misiteli, Si'iSi'i, Tahi Moana 2, TELVE, Tow Cam, Tu'i Malila, Vai Lili, Volcano O, White Church
Manus Basin	8	0	0.00	535–2500	DESMOS Cauldron, PACMANUS field, Solwara 11, Solwara 13, Solwara 17, SuSu Knolls, Vienna Woods, Vienna Woods; Hydrothermal Field 4
Mariana Arc and Trough	21	2	0.10	55–3676	Daikoku volcano, East Diamante volcano, Esmeralda Bank, Forecast, Kasuga 2 Seamount, Kasuga 3 Seamount, Maug Caldera, Minami-Hiyoshi submarine volcano, Nikko volcano, Northwest Eifuku, Northwest Rota-1 volcano, Ruby, Seamount X, TOTO Caldera, West Rota volcano, 13 N Ridge Site, Alice Springs Field, Mariana Mounds, Mariana Trough; unnamed, Pika, Snail
North East-Pacific Rise	22	1	0.05	2000–2950	AHA Field, EPR; 10 02'N, EPR; 10 44.6'N, EPR; 11 17'N, EPR; 11 24'N, EPR; 11 42'N, EPR; 13 N, EPR; 13 N; Marginal High, EPR; 21 N, EPR; 3.9 N offset, EPR; 8 38'N, EPR; 9 17'N, EPR; 9 30'N, EPR; 9 33'N, EPR; 9 40'N, EPR; 9 47'N, EPR; 9 50'N, Feather Duster, Medusa, Mounds and Microbes, Red Seamount, Teotihuacan
Okinawa Trough	11	5	0.45	30–1850	Iheya Ridge, Irabu Knoll, Izena Cauldron, Kueishan Island, Kueishan Island; offshore, Minami-Ensei Knoll, Natsushima 84-1 Knoll, North Knoll; Iheya Ridge, SPOT; Hatoma Knoll, SPOT; Yonaguni Knoll IV, Yoron Hole
South East-Pacific Rise	27	2	0.07	2064–3050	Animal Farm, EPR; 1.4 S; off-axis, EPR; 11 18'S, EPR; 14 S, EPR; 17 12'S, EPR; 17 34'S, EPR; 17 44'S, EPR; 18 10'S, EPR; 18 15'S, EPR; 18 26'S, EPR; 18 32'S, EPR; 2 S, EPR; 20 06'S, EPR; 21 25'S, EPR; 23 30'S, EPR; 23 50'S, EPR; 26 10'S, EPR; 26.5 S, EPR; 7 25'S, EPR; Ridge 1; 20 40'S propaging rift, EPR; Ridge 3; 20 40'S propaging rift, Nolan's Nook, Pito Seamount, Rapa Nui, Rehu-Marka, Saguaro Field, Stealth
Tonga Arc	7	0	0.00	210–2600	Mata Fitu, Mata Tolu, Monowai Caldera, Tonga Arc; Volcano 1, Tonga Arc; Volcano 19, Tonga Arc; Volcano P, West Mata submarine volcano

## Results and Discussion

A list of all recently known species of Alvinocaridid vent shrimps is presented in Table 2. The shrimps represent eight genera and 26 species. Most of the Alvinocaridid species inhabit the Pacific (54% species) and Atlantic (38%) Oceans. Only two species (8%) are recorded from the Indian Ocean, which may be a result of less exploration of this area.

Most species listed in Table 2 (22 of 26) were reported exclusively from hydrothermal vents. A single species, *A. longirostris*, was found both in hot vents (Kermadec fault, [9]) and cold seeps (Sagami Bay, Japan, [28]–[29]. Three species (*Alvinocaris methanophyla, A. muricola*, and *A. stactophyla*) were reported from cold seeps only. These species may be found in hot vents in the future (as occurred with *A. longirostris*), but for now they are excluded from our analyses.

While analyzing the species ranges for the obligate vent fauna, Mironov et al. [30] recognized three types of species ranges: (1) local, (2) regional, and (3) transoceanic ranges. A range is local type if the species has been recorded so far from a single hydrothermal field. A regional type of distribution represents cases where a species has been reported from more than one hydrothermal vent field within a large geographic region (e.g. Eastern Pacific, Western Pacific and the Mid-Atlantic Ridge). Species inhabiting at least two large geographic regions are classified as transoceanic in range type.

**Figure 2:**
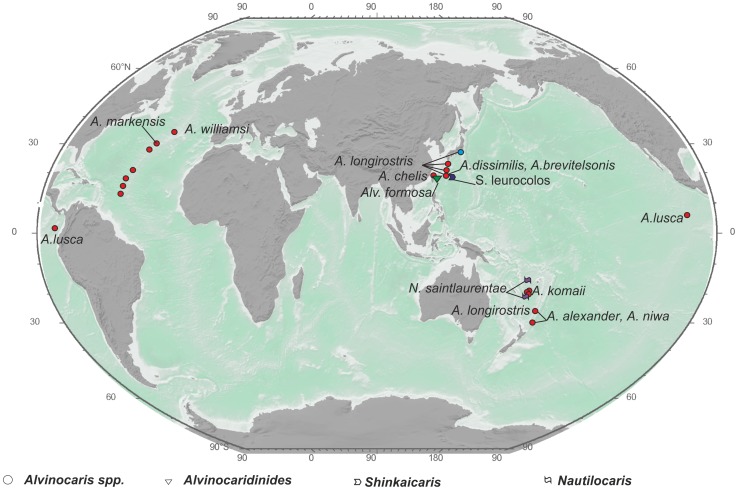
Distribution of the genera *Alvinocaris*, *Alvinocaridinides*, *Shinkaicaris*, and *Nautilocaris*. The same symbol shape indicates the same genus, and the same symbol color indicates the same species.

**Figure 3:**
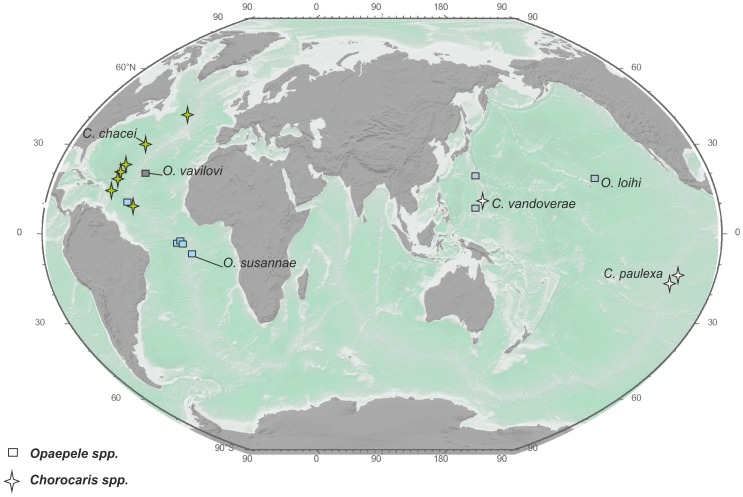
Distribution of the genera *Rimicaris* and *Mirocaris*. The same symbol shape indicates the same genus, and the same symbol color indicates the same species.

**Figure 4:**
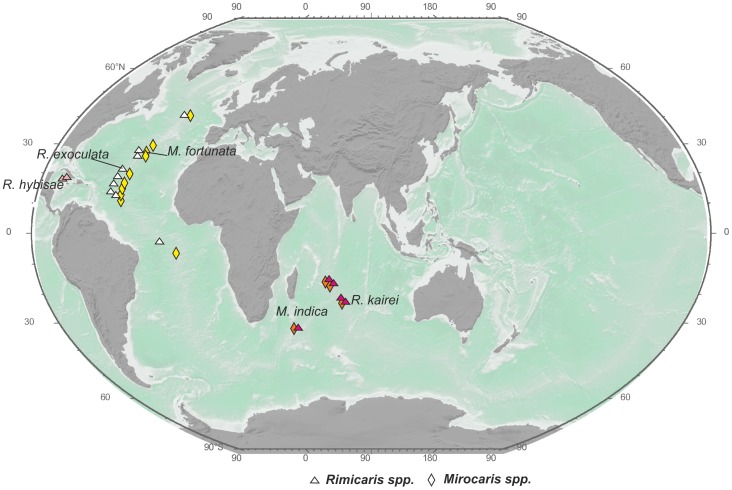
Distribution of the genera *Chorocaris* and *Opaepele*. The same symbol shape indicates the same genus, and the same symbol color indicates the same species.

For vent shrimps we observe two types of species ranges. Most species of the genera *Alvinocaris, Opaepele*, and *Chorocaris* along with the monotypic genera *Nautilocaris, Shinkaicaris*, and *Alvinocaridinides* exhibit local ranges (Fig. 2–4).

Regional-type species ranges (Fig. 2–4) are typical for Atlantic vent shrimps and for *Alvinocaris longirostris*. Ranges in this category include 3–6 hydrothermal sites mostly along the Mid-Atlantic Ridge.

As difference in sampling efforts may contribute to species distributions being classified as "local", we tried to compare sampling efforts using data in the Atlantic, Pacific, and Indian Oceans. Table 3 shows that Alvinocaridid vent shrimps occur in most geographic areas with active vents. The proportion of active vents in each region inhabited by Alvinocaridid shrimps varies from 0 (South Ocean, Juan de Fuca Ridge, Manus Basin, Tonga Arc) to 0.4–0.5 (Mid-Atlantic Ridge, Central Pacific, Okinawa Trough) and even to 0.8 (Indian Ocean). The average value is 0.22±0.06 (n = 15).

The correlation between the number of sites explored and the number of sites with Alvinocaridid shrimp records in the same area were analyzed. The Spearman correlation coefficient is 0,25 and P-level is 0,36, indicating a low relation between these parameters. We therefore suggest that differences in sampling efforts within various geographic regions do not significantly affect our conclusions.

Table 3 also indicates that all regions with vent shrimp records include vent sites with similar depth ranges, from shelf to ca. 3–5 thousand meters (except Kermadec Arc and Okinawa Trough where maximal depths are slightly less than 2 thousand meters). That means that the depth factor may not significantly control the types of ranges exhibited by species.

This is remarkable that the local type of species ranges is characteristic for the Pacific Ocean, while the regional-type species ranges are usual for the Atlantic. Most of the Atlantic vent sites occur within a long narrow rift valleys, which is absent in the eastern Pacific Ocean, and we suggest that the global biogeographic difference between eastern Pacific and Atlantic Oceans might be influenced by such geomorphological differences. Indeed, the presence of a large number of species with regional species ranges is characteristic for the rift valleys in the Atlantic Ocean and the Okinawa Trench in the western Pacific. Both areas are similar in having long, narrow, and deep bottom channels. Regional species ranges were also reported for other Atlantic vent animals: on average, the local type of distribution is reported for 75% of the obligate vent species in all oceans [19], while for the Atlantic Ocean this value is 43% [31].

**Figure 5:**
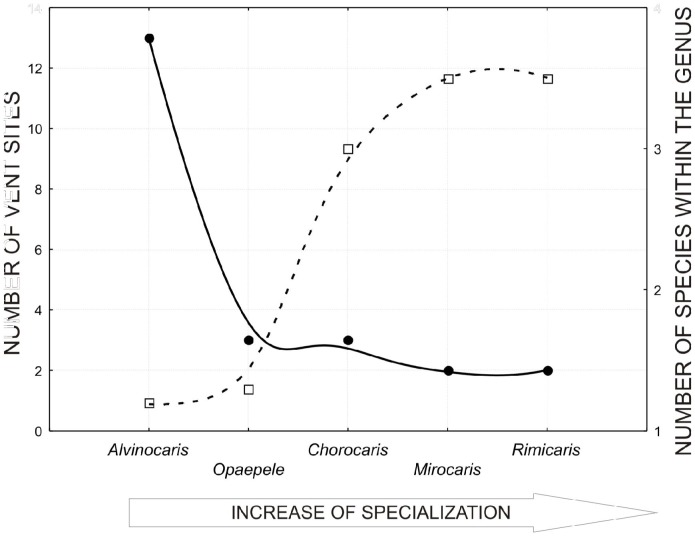
A - Mid-Atlantic Ridge, spreading rate 2.5 cm/year; B – East-Pacific Rise, full spreading rate 15 cm/year.

The Mid-Atlantic Ridge has a low spreading rate (ca 5 cm per year). The central part of rift valley is characterized by tectonic and volcanic activity (Fig. 5 A, B); it is the area where the vent communities develop. On average, the rift valley is about 12.5 km wide and 2.8 km deep [32]. The buoyant plume of hydrothermal vent fields cannot go beyond the internal rift and spread along the ridge axis [33]. The buoyant plume carries dispersal stages of shrimps from vent sites to the water column at the level of buoyant plume, approximately 200–300 m above the hydrothermal source [34]. Thus, species spread along the MAR and may colonize a number of hydrothermal fields. The rift valley may therefore serve as a corridor channeling the dispersal of a vent animal's larvae along the valley (Fig. 5A) without considerable loss of individuals.

**Figure 6:**
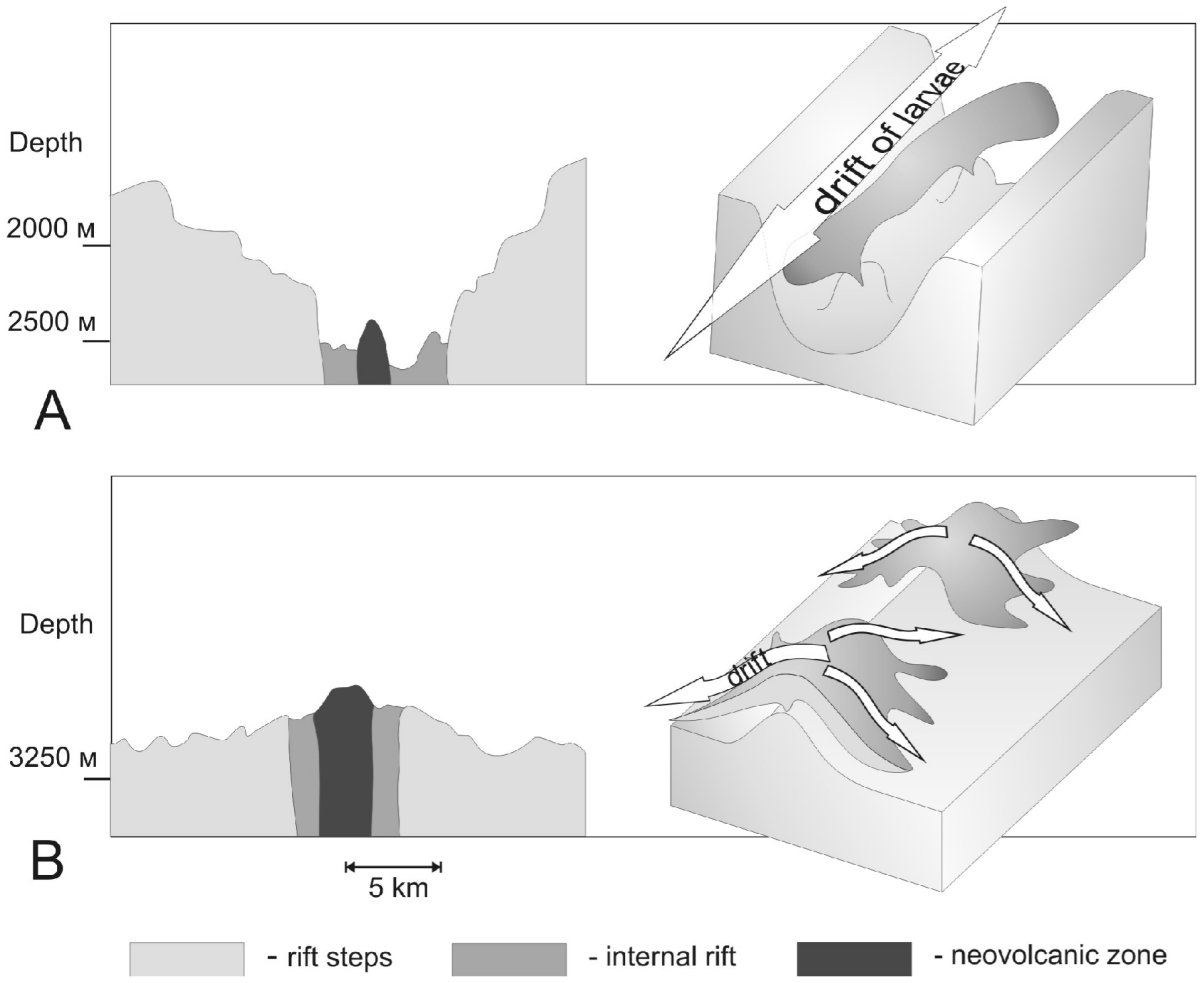
Relation between specialization, speciation and average number of sites inhabited by the species within the genus. Trend lines are made with use of distance weighted least squares analyses. These are just trends, not statistically significant effects. Specialization grows from left to right. Solid line and closed circles correspond to left y-axis. Dotted line and open squares correspond to right y-axis.

The spreading rate of the East Pacific Rise is much greater (15 cm per year) than that of the Mid-Atlantic Ridge. The valley is the apical part of the ridge and the hydrothermal plumes rise above the flanking edges [35]–[36]. Under these circumstances, deep water flows take the dispersal stages out of the rift valley, thus reducing the chance to settle at the neighboring vent fields (Fig. 6B).

The type of recruitment is reflected in the type of the species ranges. The presence of the dispersal corridor in the Atlantic Ocean may provide extensive gene exchange between populations inhabiting neighboring vent and prevent geographic isolation and speciation. In this case, we observe elongated species ranges of regional type along the Mid-Atlantic Ridge. Conversely, the absence of such a dispersal corridor in the eastern Pacific Ocean may lead to the significant loss larvae, considerable geographic isolation and higher speciation. In this case, we observe local species ranges.

Here we are faced with 2 problems:

1. There are vent taxa such as the siboglinid polychaetes that show a regional distribution along the EPR, in contrast to Alvinocaridid shrimp. A possible explanation for this pattern may be related to differences in the fecundity of these taxa and their levels of gene flow between neighbouring vent fields. At present, we do not have estimates of lifetime fecundity for any siboglinid [37]. Observations on spawning by a group of 155 females showed that a large numbers of oocytes were being spawned each day, but it was impossible to state whether any particular female spawned each day or how much. However, the average spawning rate of oocytes per female per day (over a 7-day period) was 335 (±130) [38]. Given that spawning lasts at least hundreds/thousands of days, lifetime fecundity for siboglinid could be of order of magnitude 10^4–5^ propagules per female.

Total fecundity of *Alvinocaris muricola* is related to female size and ranges between 1432 to 5798 embryos [39], e.g. 1–2 orders of magnitude less than that suggested for siboglinid polychaetes. Total abundances of alvinocaridid shrimp and siboglinid polychaetes at vent fields are difficult to estimate, but abundances of siboglinid species may be at least as high as that of alvinocaridid species. If so, the gene flow between neighbouring fields for siboglinid species could be 1–2 orders of magnitude higher than that of alvinocaridid species, if we assume comparable larval duration and mortality. This difference in potential gene flow could prevent isolation and maintain the regional type of species range for the siboglinids in the eastern Pacific. New data about the fecundity, population densities, larval mortality, and gene flow, for example from the use of molecular methods along with modeling of water mass advection, may test some of the assumptions in this overall hypothesis.

1. Fundamentally, can a "local" distribution be "real": how can a species endemic to an ephemeral environment such as hydrothermal vents only be present at only one vent field? When venting inevitably ceases at a vent field, offspring of that population must have colonised neighbouring vent fields for the species to persist. Along the MAR during monitoring of vent fields between 1994 and 2005 [26], we recorded different morphs of *Alvinocaris* (identified as species 1–6). However, final examination of hundreds of specimens (based on morphology) and statistical analysis have proven that all individuals represented a single highly diverse species [26]. At any time we observed different populations moving along the MAR. The channeling effect of the MAR rift valley may have promoted panmixia of the population through the region.

Conversely, at the EPR, lack of the channeling effect does not prevent speciation and may lead to appearance of similar related species each inhabiting 1–2 neighboring vent fields. We also acknowledge the additional possibility that a perceived "local" pattern may just be a result of uneven sampling effort so far.

With new information about composition and distribution of vent shrimps, we can consider whether these parameters are related to specialization to vent habitats demonstrated by different Alvinocaridid genera. Specialization to the vent environment in adult forms increases in the string *Alvinocaris* - *Opaepele* - *Chorocaris* - *Mirocaris – Rimicaris* [23]. This appears to lead to two effects: (1) increase of average number of vent fields inhabited by the one species of the genus and (2) decrease of species number within the genus (Fig. 6). The less specialized genus (*Alvinocaris*), with least number of adaptations to vent environments in adult form, was found to be much more speciose than the specialized genera including 2–3 species each (*Mirocaris, Rimicaris*). The leap in specialization occurs between *Alvinocaris* and all other genera of Alvinocarididae both morphologically (significantly modified characters) and ecologically (harbouring exosymbionts). It is here that we find difference in number of species within the genus (>10 in *Alvinocaris* and 2–3 each in the other genera). Each species within less specialized genera (*Opaepele*, *Chorocaris*) inhabits one or two vent fields, whilst each species of the most specialized species occurs in numerous vent sites. Genera with intermediate specialization demonstrate intermediate patterns.

The analyses of palaeontological data and living marine mollusks indicate that proportion of monotypic genera may provide an index of the genus origination rate [40]. Since most of vent shrimp genera are nearly monotypic and including two or three very similar species, we may suggests that the genus origination rate (and thus the speciation rate) within the group is high.

Analysis of the global biogeography of vent shrimps provides the clues to understand what factors drive and shape the distribution of animals under extreme environmental conditions. One possible factor, as explored here, is the influence of ridge axis geomorphology on larval dispersal. A second possible factor is the specialization to extreme biotopes that may leads to (1) extension of the species range and (2) reducing of species number within the genus, to the limit of monophyly.

**Figure 7:**
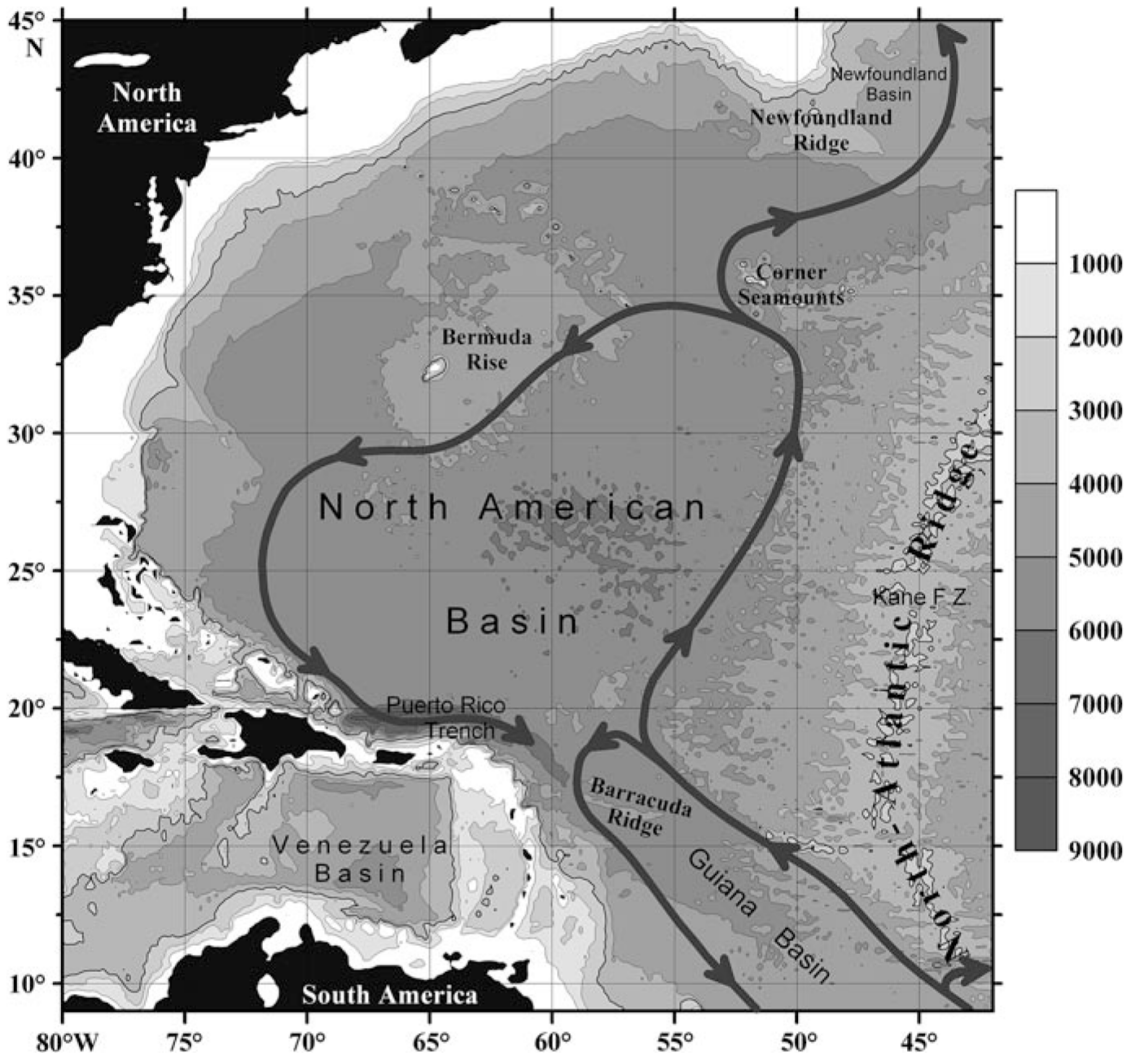
Circulation of Antarctic Bottom Water (Lower Circumpolar Water) in the Central and North Atlantic [41].

Finally, a third possible factor may be global circulation patterns acting over long periods of time: for example, near-bottom circulation in the deep Atlantic. Information about this circulation is scant, but available data indicate that it is dominated by Antarctic Bottom Water, flowing to the North American Basin after passing the Equatorial Channel and Guiana Basin [41]. Antarctic Bottom Water propagates mainly near the western slope of the Mid-Atlantic Ridge [42]–[43] and the circulation in the basin is cyclonic [43]–[45] (Fig. 7). Near-bottom meridional transport along with channeling effect is a unique feature of the Atlantic ocean that could also contribute to long regional species ranges within this area. Morphological analyses [26], [46] revealed fast population changes during 1994–2005, gene flow (measured by morphology) being apparently directed northward, coaxially with the main stream of Antarctic Bottom Water.

We may expect that species ranges from active vents in the South Atlantic are also of regional type and that any further discovered sites will appear to be populated by shrimp fauna similar to those of the North and Central Atlantic.

The Antarctic Circumpolar Current may be another factor influencing global shrimp distribution and preventing their occurrence in the Southern Ocean. According to mitochondrial cytochrome oxidase subunit analyses, vent shrimps radiated in the Miocene (less than ∼20 Myr; [47]) and since then have been distributed worldwide except the Southern Ocean. Indeed, recent description of fauna associated with high-temperature hydrothermal vents on the East Scotia Ridge in the Southern Ocean indicate an absence of Alvinocaridid shrimps [3]. But it is not just Alvinocaridid shrimp that are excluded from the Southern Ocean vents seen so far: also Bathymodiolid mussels, and indeed any vent taxa with planktotrophic larvae. This is more likely to be a result of "Thorsen's Rule", given the productivity regime of polar regions, than it is to be a result of ACC as a hydrographic barrier. Other non-planktotrophic vent taxa have managed to colonise the region arguably within the period that the ACC has been active (e.g. Kiwidae crabs; [48]). In addition, planktotrophic larvae appear to be rare among the taxa present at Arctic vents (e.g. [49]), also consistent with "Thorsen's Rule".
